# Integrating chemical and mechanical signals in neural crest cell migration

**DOI:** 10.1016/j.gde.2019.06.004

**Published:** 2019-08

**Authors:** Adam Shellard, Roberto Mayor

**Affiliations:** Department of Cell and Developmental Biology, University College London, Gower Street, London WC1E 6BT, UK

## Abstract

Neural crest cells are a multipotent embryonic stem cell population that migrate large distances to contribute a variety of tissues. The cranial neural crest, which contribute to tissues of the face and skull, undergo collective migration whose movement has been likened to cancer metastasis. Over the last few years, a variety of mechanisms for the guidance of collective cranial neural crest cell migration have been described: mostly chemical, but more recently mechanical. Here we review these different mechanisms and attempt to integrate them to provide a unified model of collective cranial neural crest cell migration.

**Current Opinion in Genetics and Development** 2019, **57**:16–24

This review comes from a themed issue on **Developmental mechanisms, patterning and evolution**

Edited by **Gáspár Jékely** and **Maria Ina Arnone**

For a complete overview see the Issue and the Editorial

Available online 13th July 2019

**https://doi.org/10.1016/j.gde.2019.06.004**

0959-437X/© 2019 The Authors. Published by Elsevier Ltd. This is an open access article under the CC BY license (http://creativecommons.org/licenses/by/4.0/).

## Introduction

A major landmark in animal evolution was the development of the neural crest, as it allowed the generation of craniofacial structures, like jaws, leading to a shift from a passive to an active mode of predation [1,2]. The neural crest is a vertebrate stem cell population that has been described as ‘the fourth germ layer’ due to its extensive contribution to several tissues during embryogenesis, including nerves, bone, connective tissue and cartilage [3]. Neural crest cells are formed during neurulation, whereby cells located at the neural plate border delaminate and undergo an epithelial-to-mesenchymal transition (EMT) [4], in which cells lose their apicobasal polarity, switch expression of adhesion proteins, and gain migratory properties [5]. The neural crest then migrates large distances across the embryo and their migratory behaviour has been likened to cancer invasion [6].

Neural crest cells have different modes of migration depending on species and location within the embryo. Some neural crest cells migrate as a mass of individuals, whereas in other cases they migrate in a highly collective manner, either as chains, groups or single sheets [7]. Collective migration is most evident in the cranial neural crest, where groups of neural crest cells move with more directionality and persistence than they do as individual cells [8]. Collective migration requires cells to be highly coordinated and cooperative, and various mechanisms have been described to explain collective migration of neural crest cells. In this review, we will outline the key processes underlying cranial neural crest cell migration, with most information coming from *Xenopus*, zebrafish and chick, before integrating them to generate an up to date unified model.

## Mechanisms of neural crest migration

**Figure 1:**
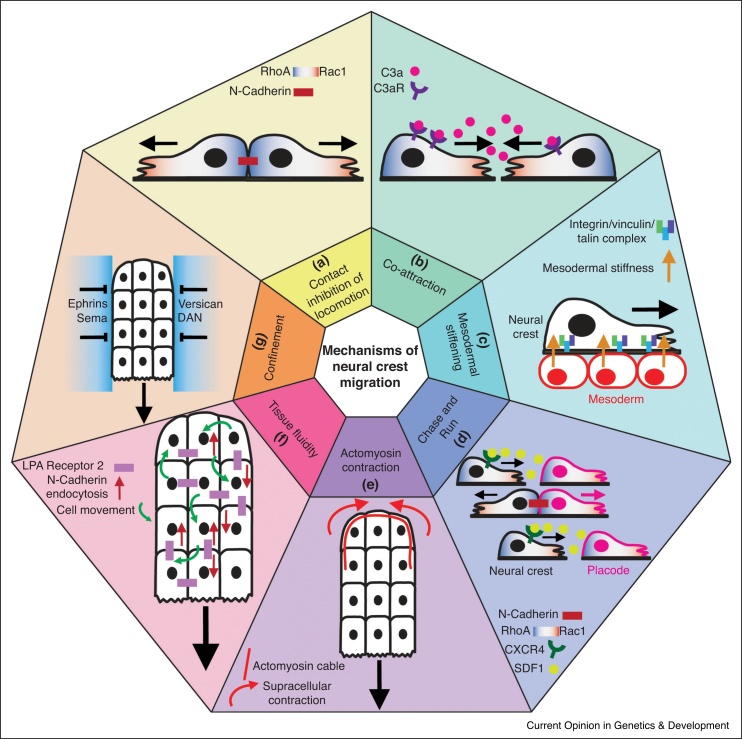
The many mechanisms of cranial neural crest cell migration. **(a)** Contact inhibition of locomotion (CIL) between neural crest cells. Upon collision of neural crest cells, a molecular cascade of signals including N-Cadherin, non-canonical Wnt/PCP signalling, EphrinB2, TBC1d24, ephrinB2, Src and FAK cause RhoA to become recruited to the contact, Rac1 to be recruited to the opposite edge and a redistribution of forces that causes the cells to repolarise and move away from each other (black arrows). In the context of a cell cluster, this means protrusions and forces are at the free-edge. **(b)** Co-attraction between neural crest cells. Neural crest cells produce the ligand C3a and its receptor C3aR, meaning cells undergo short-range chemotaxis to each other, helping to maintain the collective. **(c)** Mesodermal stiffening. Increased density of the mesoderm causes it to stiffen, which is sensed by an integrin/vinculin/talin complex in neural crest cells. This causes an upregulation in N-Cadherin and downregulation in E-Cadherin that triggers neural crest migration. **(d)** ‘Chase and run’. Neural crest cells express the CXCR4, the cognate receptor for the chemokine SDF1, which is produced by the placodal cells. Neural crest therefore ‘chase’ placodal cells by chemotaxis. Upon engagement of the two cell types, N-Cadherin, non-canonical Wnt/PCP, EphB4, EphrinB2 and TBC1d24 signalling triggers heterotypic CIL between the clusters that redistributes Rac1 and RhoA such that the neural crest ‘run’ away from the placode. ‘Chase and run’ results in the co-migration of neural crest and placodal cells. **(e)** Rear actomyosin contraction. Edge cells of the neural crest cluster have a continuous actomyosin cable. During chemotaxis, SDF1 inhibits actomyosin contraction at the front of the cluster but not at the rear. Rear contraction causes cells to intercalate that ultimately drives the cluster forward. **(f)** Tissue fluidity. LPAR2-dependent endocytosis of N-Cadherin ensures adhesions and causes constant remodelling of cell junctions, allowing cells to exchange positions. This makes the cluster to behave like a fluid. **(g)** Confinement. Extracellular signalling factors, such as ephrins, semaphorins and DAN, and extracellular matrix components, such as versican, provide inhibitory signals between the neural crest streams to the neural crest, which repels them from entering this environment.

Supracellular polarity, which refers to polarity across the whole cell cohort, is essential for migration of cell groups [9,10]. Most evidence supports the idea that collective migration, and supracellular polarity, manifests from contact inhibition of locomotion (CIL), which is the phenomenon by which colliding cells repolarise and move away from the site of cell–cell contact (Figure 1a) [11]. Accordingly, CIL is essential for cranial neural crest migration in *Xenopus*, zebrafish and chick [12,13^•^]. For a cell group, the consequences of CIL are that cell protrusions, focal adhesions and traction forces are generated at the free-edge, cryptic protrusions are inhibited, and intercellular tension is reduced [12,14,15^••^]. Thus, inner and outer cells have different mechanical properties [16].

It has been suggested that CIL is not required for some cranial neural crest cell populations, like in the chick [17] and that cell protrusions become progressively refined to the direction of migration by an unknown mechanism. However, the conclusion that chick cranial neural crest cells do not undergo CIL is based on analysis of cells that are already part of a group, where is impossible to analyse cell–cell collisions, the hallmark of CIL. Moreover, it has recently been shown that CIL can contribute to collective migration in confluent cell monolayers, like in the case of the *Drosophila* follicular epithelium, where protrusions all face the same direction [18]. Furthermore, in separate studies, CIL has been shown in both the chick cranial and trunk neural crest [13^•^,19^••^], as well as in *Xenopus* and zebrafish cranial neural crest [12,19^••^].

Another alternative to CIL-dependent collective migration is the idea that leader and follower cells are distinct subpopulations, and movement is based on leaders guiding the group forward, and trailing cells following them via the guidance of an unknown signal. This was inferred from genetic expression data in chick that suggests leader and follower cranial neural crest cells may have distinct unique transcriptional signatures [20^•^,^21^]. However, it has been demonstrated in the cranial neural crest of *Xenopus*, zebrafish and chick embryos that follower and leader cells have the capacity to take on each other’s roles, and frequently exchange positions [19^••^,22^••^,^23^,^24^].

Mechanistically, CIL in *Xenopus* and zebrafish depends on the polarised activity of the Rho GTPases, Rac1 and RhoA (Box 1 ). PCP signalling localises RhoA to sites of cell contact [12], whereas the adhesion protein N-Cadherin inhibits Rac1 activity locally, and in turn activates Rac1 at the free-edge [8]. Thus, cells establish a contact-dependent intracellular Rac/Rho gradient, with RhoA being activated at the contact and Rac1 at the free edge, leading to formation of cell protrusions at the free edge, and cells migrating into the free space. Engagement of N-Cadherin-dependent cell–cell adhesions between *Xenopus* neural crest cells results in recruitment of Src and FAK, which leads to disassembly of cell-matrix adhesions, and to a build-up of tension across the cell–cell contact that is necessary to drive separation [15^••^]. Thus, CIL involves a redistribution of adhesive forces [14,15^••^].

**Box 1:** Key molecules of cranial neural crest cell migration

Protein	What is it?	Function in the neural crest	References
RhoA	Small GTPase	Accumulates at sites of cell–cell contact to mediate CIL between neural crest cells	[12]
N-Cadherin	Cell–cell adhesion molecule	N-Cadherin is expressed by the neural crest and mediates cell–cell adhesion. It also contributes to neural crest cell-neural crest cell CIL via its inhibition of Rac1 near the cell contact. It likewise mediates CIL between the neural crest and placodal cells.	[14,23]
Rac1	Small GTPase	Rac1 is activated away from sites of cell contact. In the case of neural crest cell groups, this means it is activated at the free-edge, promoting the formation of cell protrusions.	[8]
Src	Non-receptor tyrosine kinase	Involved in the neural crest’s formation and disassembly of focal adhesions, including during CIL	[15^••^]
FAK	Focal adhesion-associated protein kinase	Involved in the neural crest’s formation and disassembly of focal adhesions, including during CIL	[15^••^]
E-Cadherin	Cell–cell adhesion molecule	E-Cadherin is expressed by the pre-migratory neural crest. During EMT, its levels are reduced, which causes a redistribution of neural crest cell forces away from sites of cell contact and toward the group’s edge.	[14,22^••^]
PDGF/PDGFR	Growth factor and its receptor	Neural crest cells express both the ligand and the receptor. Autocrine PDGF signalling regulates N-Cadherin.	[27^•^]
Cx43	Connexin-43, a gap junction protein	Neural crest cells express Cx43. The carboxy tail of Cx43 interacts with the basic transcription factor-3 to directly regulate N-Cadherin expression after binding to its promoter.	[28^••^]
EphrinB2/EphB4	Membrane-bound ligand (ephrin) and receptor (Eph)	In the surrounding extracellular matrix, ephrin signalling to the neural crest prevents it from moving into exclusion zones. Signalling between neural crest cell and placodal cells, it controls CIL and collective chemotaxis.	[29^••^]
TBC1d24	Rab35-GTPase activating protein	Interacts with EphrinB2 to control CIL between neural crest cells.	[29^••^]
C3a/C3aR	Complement component, C3a, and its receptor, C3aR	Expressed by the neural crest to promote paracrine short-range chemotaxis (co-attraction), preventing neural crest dispersion.	[34]
Integrin/vinculin/talin	Form part of the cell-matrix adhesion complex.	The neural crest express α5β1 integrin, which interacts with fibronectin in the surrounding extracellular matrix. Vinculin and talin proteins complex with integrin intracellularly and are required for the neural crest’s mechanical response to extracellular stiffness.	[35^••^,^43^]
HIF1α	Transcription factor	HIF1α is expressed by the neural crest and controls the expression of a master regulator of EMT, Twist.	[47]
SDF1	Chemokine	SDF1 is expressed by the placodal cells. Neural crest expresses its receptor, CXCR4, and undergo chemotaxis toward the signal (placodes).	[22^••^,^8^]
VEGF	Growth factor	Neural crest undergo chemotaxis to VEGF.	[38,24]
FGF8	Growth factor	Neural crest undergo chemotaxis to FGF8.	[39]
LPAR2	G protein-coupled receptor	Neural crest cells express LPAR2. LPA signalling results in endocytosis of N-Cadherin, which increases tissue fluidity.	[23]
Versican	Extracellular matrix proteoglycan	Versican is found in the exclusion boundaries between neural crest streams. It inhibits neural crest migration into these zones, and simultaneously promotes neural crest migration within the stream by enhancing its confinement.	[42]
DAN	BMP antagonist	DAN is expressed in the mesoderm and inhibits neural crest cell migration.	[44^•^]
GSK3	Serine/threonine protein kinase	GSK3 is a central regulator of signalling in the neural crest. It controls key regulators of migration, including Rac1, lamellipodin and FAK.	[50^••^]

N-Cadherin is therefore a central regulator of CIL, and alterations in its levels, as well as those of other cadherins, including E-Cadherin, cadherin-11 and protocadherins, can exert substantive effects on cranial neural crest migration in *Xenopus* [8,25,26]. For instance, the switch of E-Cadherin to N-Cadherin during EMT is essential for the acquisition of CIL in migratory neural crest [14]. The importance of N-Cadherin regulation is illustrated by the many levels at which it is controlled. *Xenopus* neural crest cells produce PDGF and express its receptor PDGFR, which regulates N-Cadherin in an autocrine manner, thereby contributing to CIL [27^•^]. At the transcriptional level, N-cadherin in the neural crest is controlled by the intracellular domain of the gap junction protein Connexin 43 (Cx43) [28^••^]. Furthermore, signals arising from the interaction between ephrinB2 and TBC1d24, a Rab35 GAP, which are both expressed by the neural crest, also controls CIL between cranial neural crest cells in *Xenopus* [29^••^]. TBC1d24 regulates E-Cadherin endocytosis, ensuring that its levels are kept low at the cell membrane, thereby permitting efficient migration [29^••^]. Although these studies use *Xenopus*, it is likely that the mechanisms are similar for other vertebrates, including mouse and chick, because many of these proteins, such as EphrinB2, have conserved expression [30,31].

CIL has the capacity to disperse a cell population [32]. The neural crest remain as a collective, in part, thanks to short-range chemotaxis (termed co-attraction) dependent on C3a, a factor of the complement cascade well known for its chemoattractant activity in the immune system [33]. All *Xenopus* neural crest cells express both the ligand C3a and its receptor C3aR (Figure 1b) [34]. This means that dispersing cells are chemoattracted back to the cell group, which has a high concentration of C3a. CIL and co-attraction must therefore be finely tuned to ensure that the cranial neural crest is maintained as a loosely associated cell population [34].

Beyond N-Cadherin’s role in CIL, its importance for adherence of the neural crest cell population is evident in the fact that the neural crest cannot migrate efficiently as individuals [8]. Apart from chemical regulation arising from neural crest cell–cell contacts, N-Cadherin is also regulated downstream of the tissue’s mechanical response to its surroundings in *Xenopus* embryos. During development, the mesoderm, which sits underneath the neural crest, becomes stiffer as a consequence of an increase in cell density [35^••^]. This mechanical change causes the neural crest of *Xenopus* to migrate (Figure 1c) seemingly thanks to activation of EMT: *in vitro*, neural crest cells disperse, upregulate N-Cadherin and downregulate E-Cadherin when cultured on stiff, but not soft, substrates [35^••^]. The mechanism by which mechanical signals are transduced to affect migration is unclear, although integrin, vinculin and talin are known to be required [35^••^]. Similarly, enteric neural crest colonisation of the gut in chick and mouse is dependent on stiffening of the surrounding mesenchyme [36]. Thus, neural crest migration is regulated not only by chemical signals but also by mechanical ones.

Cranial neural crest cells migrate by chemotaxis toward sources of chemoattractant *in vivo* [37], such as SDF1 in *Xenopus* and zebrafish [8], VEGF in chick [24,38], and FGF8 in mouse [39], which are essential for migration *in vivo* [8,38]. SDF1, the most well-characterised chemoattractant of the neural crest, activates Rac1 in cells at the front of the group, enhancing and stabilising front cell protrusions and focal adhesions [8]. SDF1 is produced by placodal cells, an embryonic cell population that together with neural crest generates the cranial nerves in the head. The interaction between neural crest and placodes mediated by SDF1 has been called ‘chase and run’ [40]. Neural crest cells ‘chase’ placodal cells by chemotaxis, and upon engagement of the two cell types, there is heterotypic CIL, meaning the placodal cells ‘run’ away from the neural crest (Figure 1d) [40]. This ‘chase and run’ behaviour results in coordinated migration of the two cell populations. Like homotypic CIL between neural crest cells, heterotypic repulsion involves PCP and N-Cadherin signalling. Moreover, placodal cells express EphB4, the ligand for ephrinB2, which is expressed in the neural crest, and this interaction also mediates the ‘run’ response when the two cell populations make contact [29^••^].

As well as enhancing front cell protrusions, SDF1 further promotes front-rear collective polarity by inhibiting contractility at the front of the cluster [22^••^]. Outer edge cells in *Xenopus* and zebrafish form a continuously linked tensile actomyosin cable, and the external SDF1 gradient polarises its mechanical activity [22^••^]. Experiments using optogenetic control of RhoA reveal that contraction at the rear of neural crest cell groups drive clusters forward by collective chemotaxis (Figure 1e); collective migration in the absence of SDF1 *in vivo* can be rescued by increasing rear contraction [22^••^]. Overall, chemical gradients polarise neural crest cell collectives and alter the group’s mechanical properties through traction and contraction forces to enable efficient forward movement.

During directional migration, cells must constantly adapt to the changing architecture and constraints through which they move. Polarised actomyosin contraction causes an anterograde flow of cells that begins with cell intercalation at the cluster rear [22^••^]. In *Xenopus* and zebrafish, cells flow forward in the middle of the cluster and are mechanically pulled rearwards at the cluster edges, due to the contraction of the supracellular actomyosin cable [22^••^]. The migration overall is like that of a fluid, in that cells can easily exchange positions [23]. This fluid-like movement is promoted by endocytosis of N-Cadherin by LPA receptor 2, which allows cells to continually remodel their cell–cell junctions (Figure 1f), and is essential for migration *in vivo* [23].

The neural crest is also responsive to surrounding chemorepellents. Inhibitory signals exist between the neural crest streams of various species, which include ephrins and class 3 semaphorins (Figure 1g) [41]. These repulsive signals prevent neural crest cells from invading non-neural crest tissue, and prevent mixing of neural crest from different streams [41]. These repulsive signals at the border of the neural crest confines the cells, thereby enhancing their migration (Figure 1g) [42]. The neural crest is sandwiched between the epidermis above it and the underlying mesoderm. Between these layers are matrix components which primarily consist of fibronectin, which is secreted by the mesoderm [43]. The neural crest require fibronectin to migrate, interacting with it via α5β1 integrins [43]. The extracellular matrix proteoglycan, versican, is expressed in between the streams, promoting directional migration of the neural crest by forming exclusion boundaries [42]. Likewise, the BMP antagonist DAN is expressed by the mesoderm in regions lateral to the chick cranial neural crest, restraining neural crest migration by moderating its speed [44^•^]. In this manner, neural crest cells are maintained at a high density, encouraging cell–cell interactions which are required for collective movement. The neural crest also express MMPs and ADAMs in various species, which help degrade and remodel the extracellular matrix and also regulate EMT [45,46].

## Integrating signals in neural crest migration

**Figure 2:**
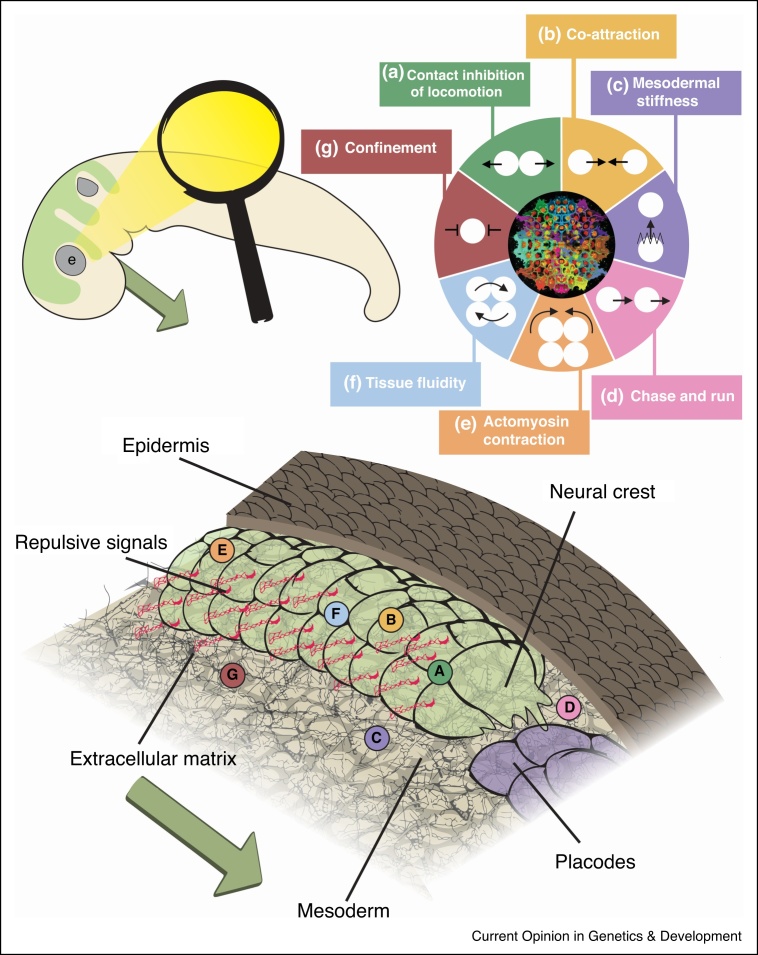
A coherent, integrated model of cranial neural crest migration *in vivo*. Top left, a vertebrate embryo. The cranial neural crest (green) migrates from dorsal regions (top/left) to the pharyngeal arches (bottom/right) in the head of the embryo. The dark green arrow indicates the direction of migration. The neural crest migrates collectively and in stereotypical streams, avoiding structures such as the eye (e). Bottom panel, cranial neural crest migration *in vivo* requires the integration of many cell-intrinsic and cell-extrinsic chemical and mechanical signals. This diagram illustrates some of the many inputs the neural crest receives which helps dictate its efficient movement. The mechanisms are lettered and correspond to the simplistic diagrammatic legend (top left). **(a)** CIL between neural crest cells (green) ensures protrusions are only formed at free edge. **(b)** Co-attraction between neural crest cells prevents dispersion. **(c)** Mesodermal (pale brown) stiffening is mechanosensed by the neural crest. **(d)** ‘Chase and run’ between the neural crest and the placode cells (purple). **(e)** Actomyosin contraction at the rear of the neural crest cell group. **(f)** Tissue fluidity promotes cell exchange and a ‘fluid’ behaviour of the neural crest. **(g)** Confinement of the neural crest between epidermis (dark brown), mesoderm and surrounding inhibitory signals (red) including extracellular matrix proteins and proteoglycans (black meshwork) and repulsive extracellular signals. Fibronectin is the substrate required for neural crest cell migration.

The integration of many signals – those from chemorepellents, chemoattractants, mechanical stiffness, extracellular matrix molecules and cell–cell adhesions – likely demands cross-talk at different levels (Figure 2). At the molecular level, N-Cadherin appears to be a central target for regulation [27^•^,28^••^,35^••^,^47^], which affects cellular and supracellular behaviour including tissue fluidity and CIL [12,23]. Diverse mechanisms, such as hypoxia and mechanical signals, regulate N-Cadherin expression in the cranial neural crest [35^••^,^47^]. Some E-Cadherin may be required for cranial neural crest migration too [48], although it remains controversial what function it has, if any.

Many signals converge on Rho GTPases – which are the main orchestrators of cell motility [49] – and the downstream targets of CIL, co-attraction, chemoattractants and chemorepellents. Furthermore, GSK3 is a central hub for regulating neural crest migration in both mouse and *Xenopus*: it is required to establish polarity, and to form lamellipodia and focal adhesions, through its regulation of FAK, Rac1 and lamellipodin [50^••^]. Signals can also converge on cell-surface receptors, as is the case for Nrp1, a co-receptor for Sema3, VEGF and PDGF. Likewise, EphrinB2 is involved in both heterotypic and homotypic CIL, binding to either EphB4 or TBC1d24 depending on its cell adhesion partner [29^••^].

This question of how different signals are integrated to regulate migration has been directly addressed recently, in *Xenopus* and chick cranial neural crest. SDF1, a chemoattractant proposed by placodal cells, and Sema3A, a repulsive signal surrounding the neural crest, act antagonistically on Rac1 activity [51^••^]. SDF1 activates Rac1 and promotes cell-matrix adhesions, whereas Sema3A has the opposite effect [51^••^]. The different distributions of SDF1 and Sema3A contribute to directional migration of the whole cell population, indicating that the time and location of such signal cross-talk is important for migration.

From a mechanical point of view, stresses from traction provide pulling forces at the cohort’s edge that are enhanced by SDF1 [8,14]. Complimentarily, actomyosin-dependent contractile forces at the rear provide pushing forces [22^••^]. These mechanical properties of neural crest cells seem to be intimately linked to cadherins. In pre-migratory *Xenopus* neural crest, which expresses high levels of E-Cadherin, more actomyosin accumulates at sites of cell–cell contact, cells have cryptic protrusions and traction forces are in the middle of the cell group [14,22^••^]. By contrast, in migratory neural crest, which express high levels of N-Cadherin and low levels of E-cadherin, contractile and traction forces are at the edge, indicating a redistribution of forces away from intercellular tension [14,22^••^]. Low intercellular tension may also contribute to the tissue’s fluidity. In addition to mechanical forces exerted by the neural crest to move, the mechanical environment is also sensed by the neural crest to regulate movement, and confinement provides a constrained environment for the neural crest and cells are mechanosensitive to the environment [23,35^••^].

How is CIL, which separates cells away from the contact [11], compatible with actomyosin contraction forces, whereby edge cells move closer to internal cells through intercalation [22^••^]? CIL allows neural crest cell clusters to migrate, but it does not specify direction. As rear cells intercalate during collective chemotaxis, they could potentially exert pushing forces on the cells in front to mechanically force them to move forward. Alternatively, intercalating cells may increase cell surface adhesion with those cells in front, thereby increasing the CIL response and causing their neighbours to move forward. If CIL is mechanoresponsive, like it is in other systems [52], it would be interesting to understand how the forces of edge neural crest cells impact CIL in central regions. Importantly, the tension transmitted across the actomyosin cable must be strong enough to cause retrograde flow around the cohort’s edge [22^••^]. This is compatible and complementary to CIL, in which edge cells would struggle to enter the middle of the cluster unless a strong force caused them to intercalate. Indeed, the middle of neural crest clusters exhibit fluid behaviour [23]. Furthermore, as edge cells near the front are pulled rearward, new cells can emerge as transient leaders. In this manner, middle cells may move forward passively while still engaging in CIL.

Many of the mechanisms of neural crest migration have cooperative effects at both the cellular level and supracellular level. CIL generates front-rear polarity in cells at the edge of the cohort, and SDF1 enhances this polarity [8,12,22^••^]. At the level of the cluster, the two mechanisms together generate the supracellular polarity needed for directional migration: CIL generates differences between the inside and outside of the cluster, and SDF1 creates front-rear differences across the cluster [8,12,22^••^]. The upstream chemical signals coordinating these behaviours ultimately lead to the polarised mechanics that are necessary for directed movement. CIL also affects the neural crest’s ability to respond to chemoattractants [29^••^], indicating direct cross-talk between these processes. This is further complicated by the fact that the same signalling molecules can control these interacting processes. For instance, PDGF not only regulates CIL but is also a chemoattractant for the neural crest [27^•^]. There is also cross-talk between CIL and co-attraction: a delicate balance between these levels is needed to ensure cells do not either disperse nor remain too compact [34]. Additionally, there must be cross-talk between signalling pathways that evoke similar behaviours. For instance, the repulsion between neural crest and placode – but not between neural crest cells – is independent of TBC1d24, indicating that both overlapping and separate mechanisms exist for homotypic and heterotypic CIL [29^••^]. Likewise, there must be interactions between the pathways downstream of C3a/C3aR and SDF1/CXCR4 to regulate the chemotactic processes. Overall, the diverse mechanisms of neural crest migration intertwine with each other at many levels.

## Future directions

Since the discovery of the neural crest 150 year ago by Wilhelm His, we have gone a long way in understanding the cellular and molecular mechanisms that explain the migration of this captivating cell type, and we are only now beginning to understand how these different mechanisms are integrated. Many interesting questions remain to be addressed. Although many of the mechanisms of neural crest migration have been described, it is unclear how cells know when to stop migrating. The neural crest stops migrating when they reach the pharyngeal arches, which may be due to a loss of chemoattractant for them to follow. By this point they have already migrated enormous distances across the embryo. The neural crest may stop moving when they reach a low enough density that cells disperse and cannot be co-attracted back. Single cells migrate far less efficiently as individuals toward chemotactic signals [8], so this would prevent further chemotaxis. Also, given the initiation of neural crest migration might be linked to the *in vivo* topology surrounding the neural crest and the way in which different signals are integrated [51^••^], migration may be ceased by a similar mechanism.

## Conflict of interest statement

Nothing declared.

## References and recommended reading

Papers of particular interest, published within the period of review, have been highlighted as:

• of special interest
•• of outstanding interest

## References

[1] Donoghue P.C.J., Graham A., Kelsh R.N. (2008). The origin and evolution of the neural crest. Bioessays.

[2] Munoz W.A., Trainor P.A. (2015). Neural crest cell evolution: how and when did a neural crest cell become a neural crest cell. Neural Crest Placodes.

[3] Dupin E., Coelho-Aguiar J.M. (2013). Isolation and differentiation properties of neural crest stem cells. Cytometry A.

[4] Theveneau E., Mayor R. (2012). Neural crest delamination and migration: from epithelium-to-mesenchyme transition to collective cell migration. Dev Biol.

[5] Nieto M.A., Huang R.Y.J., Jackson R.A., Thiery J.P. (2016). EMT: 2016. Cell.

[6] Kerosuo L., Bronner-Fraser M. (2012). What is bad in cancer is good in the embryo: importance of EMT in neural crest development. Semin Cell Dev Biol.

[7] Theveneau E., Mayor R. (2011). Can mesenchymal cells undergo collective cell migration? The case of the neural crest. Cell Adhes Migr.

[8] Theveneau E., Marchant L., Kuriyama S., Gull M., Moepps B., Parsons M., Mayor R. (2010). Collective chemotaxis requires contact-dependent cell polarity. Dev Cell.

[9] Shellard A., Mayor R. (2019). Supracellular migration: beyond collective cell migration. J Cell Sci.

[10] Mayor R., Etienne-Manneville S. (2016). The front and rear of collective cell migration. Nat Rev Mol Cell Biol.

[11] Stramer B., Mayor R. (2017). Mechanisms and in vivo functions of contact inhibition of locomotion. Nat Rev Mol Cell Biol.

[12] Carmona-Fontaine C., Matthews H.K., Kuriyama S., Moreno M., Dunn G.A., Parsons M., Stern C.D., Mayor R. (2008). Contact inhibition of locomotion in vivo controls neural crest directional migration. Nature.

[13•] Li Y.W., Vieceli F.M., Gonzalez W.G., Li A., Tang W.Y., Lois C., Bronner M.E. (2019). In vivo quantitative imaging provides insights into trunk neural crest migration. Cell Rep.

[14] Scarpa E., Szabo A., Bibonne A., Theveneau E., Parsons M., Mayor R. (2015). Cadherin switch during EMT in neural crest cells leads to contact inhibition of locomotion via repolarization of forces. Dev Cell.

[15••] Roycroft A., Szabo A., Bahm I., Daly L., Charras G., Parsons M., Mayor R. (2018). Redistribution of adhesive forces through Src/FAK drives contact inhibition of locomotion in neural crest. Dev Cell.

[16] Blaue C., Kashef J., Franz C.M. (2018). Cadherin-11 promotes neural crest cell spreading by reducing intracellular tension-mapping adhesion and mechanics in neural crest explants by atomic force microscopy. Semin Cell Dev Biol.

[17] Genuth M.A., Allen C.D., Mikawa T., Weiner O.D. (2018). Chick cranial neural crest cells use progressive polarity refinement, not contact inhibition of locomotion, to guide their migration. Dev Biol.

[18] Stedden C.G., Menegas W., Zajac A.L., Williams A.M., Cheng S.Q., Ozkan E., Horne-Badovinac S. (2019). Planar-polarized semaphorin-5c and Plexin A promote the collective migration of epithelial cells in Drosophila. Curr Biol.

[19••] Richardson J., Gauert A., Montecinos L.B., Fanlo L., Alhashem Z.M., Assar R., Marti E., Kabla A., Hartel S., Linker C. (2016). Leader cells define directionality of trunk, but not cranial, neural crest cell migration. Cell Rep.

[20•] Morrison J.A., Mclennan R., Wolfe L.A., Gogol M.M., Meier S., Mckinney M.C., Teddy J.M., Holmes L., Semerad C.L., Box A.C. (2017). Single-cell transcriptome analysis of avian neural crest migration reveals signatures of invasion and molecular transitions. eLife.

[21] Mclennan R., Schumacher L.J., Morrison J.A., Teddy J.M., Ridenour D.A., Box A.C., Semerad C.L., Li H., Mcdowell W., Kay D. (2015). Neural crest migration is driven by a few trailblazer cells with a unique molecular signature narrowly confined to the invasive front. Development.

[22••] Shellard A., Szabo A., Trepat X., Mayor R. (2018). Supracellular contraction at the rear of neural crest cell groups drives collective chemotaxis. Science.

[23] Kuriyama S., Theveneau E., Benedetto A., Parsons M., Tanaka M., Charras G., Kabla A., Mayor R. (2014). In vivo collective cell migration requires an LPAR2-dependent increase in tissue fluidity. J Cell Biol.

[24] McLennan R., Schumacher L.J., Morrison J.A., Teddy J.M., Ridenour D.A., Box A.C., Semerad C.L., Li H., McDowell W., Kay D. (2015). VEGF signals induce trailblazer cell identity that drives neural crest migration. Dev Biol.

[25] Becker S.F.S., Mayor R., Kashef J. (2013). Cadherin-11 mediates contact inhibition of locomotion during *Xenopus* neural crest cell migration. PLoS One.

[26] Mccusker C., Cousin H., Neuner R., Alfandari D. (2009). Extracellular cleavage of cadherin-11 by ADAM metalloproteases is essential for *Xenopus* cranial neural crest cell migration. Mol Biol Cell.

[27•] Bahm I., Barriga E.H., Frolov A., Theveneau E., Frankel P., Mayor R. (2017). PDGF controls contact inhibition of locomotion by regulating N-cadherin during neural crest migration. Development.

[28••] Kotini M., Barriga E.H., Leslie J., Gentzel M., Rauschenberger V., Schambon A., Mayor R. (2018). Gap junction protein Connexin-43 is a direct transcriptional regulator of N-cadherin in vivo. Nat Commun.

[29••] Yoon J., Hwang Y.S., Lee M., Sun J., Cho H.J., Knapik L., Daar I.O. (2018). TBC1d24-ephrinB2 interaction regulates contact inhibition of locomotion in neural crest cell migration. Nat Commun.

[30] Adams R.H., Diella F., Hennig S., Helmbacher F., Deutsch U., Klein R. (2001). The cytoplasmic domain of the ligand ephrinB2 is required for vascular morphogenesis but not cranial neural crest migration. Cell.

[31] Mellott D.O., Burke R.D. (2008). Divergent roles for Eph and Ephrin in avian cranial neural crest. BMC Dev Biol.

[32] Davis E.M., Trinkaus J.P. (1981). Significance of cell-to-cell contacts for the directional movement of neural crest cells within a hydrated collagen lattice. J Embryol Exp Morphol.

[33] Ricklin D., Hajishengallis G., Yang K., Lambris J.D. (2010). Complement: a key system for immune surveillance and homeostasis. Nat Immunol.

[34] Carmona-Fontaine C., Theveneau E., Tzekou A., Tada M., Woods M., Page K.M., Parsons M., Lambris J.D., Mayor R. (2011). Complement fragment C3a controls mutual cell attraction during collective cell migration. Dev Cell.

[35••] Barriga E.H., Franze K., Charras G., Mayor R. (2018). Tissue stiffening coordinates morphogenesis by triggering collective cell migration in vivo. Nature.

[36] Chevalier N.R., Gazguez E., Bidault L., Guilbert T., Vias C., Vian E., Watanabe Y., Muller L., Germain S., Bondurand N. (2016). How tissue mechanical properties affect enteric neural crest cell migration. Sci Rep.

[37] Shellard A., Mayor R. (2016). Chemotaxis during neural crest migration. Semin Cell Dev Biol.

[38] Mclennan R., Teddy J.M., Kasemeier-Iulesa J.C., Romine M.H., Kulesa P.M. (2010). Vascular endothelial growth factor (VEGF) regulates cranial neural crest migration in vivo. Dev Biol.

[39] Creuzet S., Schuler B., Couly G., Le Douarin N.M. (2004). Reciprocal relationships between Fgf8 and neural crest cells in facial and forebrain development. Proc Natl Acad Sci U S A.

[40] Theveneau E., Steventon B., Scarpa E., Garcia S., Trepat X., Streit A., Mayor R. (2013). Chase-and-run between adjacent cell populations promotes directional collective migration. Nat Cell Biol.

[41] Theveneau E., Mayor R. (2012). Neural crest migration: interplay between chemorepellents, chemoattractants, contact inhibition, epithelial-mesenchymal transition, and collective cell migration. Wiley Interdiscip Rev Dev Biol.

[42] Szabo A., Melchionda M., Nastasi G., Woods M.L., Campo S., Perris R., Mayor R. (2016). In vivo confinement promotes collective migration of neural crest cells. J Cell Biol.

[43] Alfandari D., Cousin H., Gaultier A., Hoffstrom B.G., Desimone D.W. (2003). Integrin alpha 5 beta 1 supports the migration of *Xenopus* cranial neural crest on fibronectin. Dev Biol.

[44•] Mclennan R., Bailey C.M., Schumacher L.J., Teddy J.M., Morrison J.A., Kasemeier-Kulesa J.C., Wolfe L.A., Gogol M.M., Baker R.E., Maini P.K., Kulesa P.M. (2017). Dan (Nbl1) promotes collective neural crest migration by restraining uncontrolled invasion. J Cell Biol.

[45] Christian L., Bahudhanapati H., Wei S. (2013). Extracellular metalloproteinases in neural crest development and craniofacial morphogenesis. Crit Rev Biochem Mol Biol.

[46] Garmon T., Wittling M., Nie S.Y. (2018). MMP14 regulates cranial neural crest epithelial-to-mesenchymal transition and migration. Dev Dyn.

[47] Barriga E.H., Maxwell P.H., Reyes A.E., Mayor R. (2013). The hypoxia factor Hif-1 alpha controls neural crest chemotaxis and epithelial to mesenchymal transition. J Cell Biol.

[48] Huang C., Kratzer M.C., Wedlich D., Kashef J. (2016). E-cadherin is required for cranial neural crest migration in *Xenopus laevis*. Dev Biol.

[49] Ridley A.J., Schwartz M.A., Burridge K., Firtel R.A., Ginsberg M.H., Borisy G., Parsons J.T., Horwitz A.R. (2003). Cell migration: integrating signals from front to back. Science.

[50••] Malagon S.G.G., Munoz A.M.L., Doro D., Bolger T.G., Poon E., Tucker E.R., Al-Lami H.A., Krause M., Phiel C.J., Chesler L., Liu K.J. (2018). Glycogen synthase kinase 3 controls migration of the neural crest lineage in mouse and *Xenopus*. Nat Commun.

[51••] Bajanca F., Gouignard N., Colle C., Parsons M., Mayor R., Theveneau E. (2019). In vivo topology converts competition for cell-matrix adhesion into directional migration. Nat Commun.

[52] Weber G.F., Bjerke M.A., Desimone D.W. (2012). A mechanoresponsive cadherin-keratin complex directs polarized protrusive behavior and collective cell migration. Dev Cell.

